# Multicenter survey on viscoelastic assays access and use in trauma in Swiss emergency departments

**DOI:** 10.1007/s00068-026-03092-w

**Published:** 2026-03-02

**Authors:** Matthieu Lyon, Timothé De Valence, Christophe A. Fehlmann

**Affiliations:** 1 Division of Emergency Medicine, Department of Acute Care Medicine, Geneva University Hospitals, 4 Rue Gabrielle-Perret-Gentil, Geneva 14, CH-1211 Switzerland; 2 Division of Anesthesiology, Department of Acute Care Medicine, Geneva University Hospitals, 4 Rue Gabrielle-Perret-Gentil, Geneva 14, CH-1211 Switzerland; 3 Department of Anaesthesiology, Pharmacology, Intensive Care and Emergency Medicine, Faculty of Medicine, University of Geneva, 4 Rue Gabrielle-Perret-Gentil, Geneva 14, CH-1211 Switzerland

**Keywords:** Viscoelastic testing, Viscoelastic assays, Trauma, Coagulopathy, ROTEM, TEG

## Abstract

**Purpose:**

Trauma is a global cause of morbidity and mortality, with early deaths frequently resulting from hemorrhage and traumatic brain injury. Prompt assessment and targeted management of bleeding and trauma-induced coagulopathy are essential in this context. Viscoelastic tests (VETs), such as TEG and ROTEM, offer advantages over conventional coagulation. Therefore, we wanted to evaluate their availability and use in Swiss emergency departments (EDs).

**Methods:**

A 16-item online survey assessing access, use, and training related to VET, as well as the use of massive hemorrhage protocols, was distributed to 52 EDs recognized by the Swiss Society of Emergency and Rescue Medicine. Twelve were level-1 trauma centers, five of which were university hospitals.

**Results:**

The response rate was 86.5% (45/52). Access to VET was reported by 44.4% of centers, while routine use in major trauma was noted in 26.7%. Most users were trauma centers. Devices were predominantly located outside the ED (e.g., laboratories or operating rooms). Over half of the centers with access to VET reported no formal training. Additionally, 51.1% of respondents indicated the absence of a dedicated massive hemorrhage protocol. Among those with protocols, most used conventional coagulation tests, and fewer included VET.

**Conclusion:**

The use of VET in Swiss EDs is limited, mainly to trauma centers, with restricted point-of-care access and heterogeneous training. Massive hemorrhage protocols are inconsistently implemented and often lack integration of VET. Key areas for improvement include expanding access beyond specialized centers, standardizing protocols and interpretation algorithms, optimizing device placement, and introducing structured training programs.

**Supplementary Information:**

The online version contains supplementary material available at https://doi.org/10.1007/s00068-026-03092-w.

## Background

Trauma is a major global health issue accounting for an estimated 4.4 million deaths annually and contributing to approximately 10% of all years lived with disability [1]. In the European Union, trauma is the leading cause of death among individuals aged 15 to 34 years [2]. Bleeding and hemorrhagic shock account for 30 to 40% of trauma-related deaths, with most bleeding-related fatalities occurring within the first 24 h [3].

Acute traumatic coagulopathy is a complex, multifactorial process affecting approximately 25–30% of severely injured patients and leading to worse outcomes, including increased blood product requirements, longer hospital stays, and higher mortality [4, 5]. The recent international guidelines on major bleeding following trauma advocate for early and repeated coagulation monitoring, along with goal-directed treatment strategies (either by conventional coagulation tests or viscoelastic testing) over empirical management [6, 7]. Viscoelastic testing’s advantages include a comprehensive overview of coagulation, the possibility of point-of-care testing, fast results (about five minutes for early results) and more sensitivity in detecting acute traumatic coagulopathy compared with conventional coagulation tests [8–11].

Switzerland is a federal state comprising 26 cantons, with a population of around 9 million and four official national languages [12]. Many health-related matters are managed at the cantonal level, resulting in considerable variation in practices nationally. No data is currently available on the access, utilization, and training related to these tests in Swiss emergency department settings.

The objectives of this study were (1) to give a first estimate of the frequency and modality of viscoelastic testing access and use in emergency departments in Switzerland (2) to examine how frequently massive hemorrhage protocols are used and to what extent they incorporate conventional coagulation tests and viscoelastic assays, and (3) to evaluate the type and level education provided by hospitals using these methods.

## Methods

We created an English-language cross-sectional online survey using Microsoft Forms. It consisted of up to 16 questions, with an additional 17th field for optional comments. The first 3 items were factual identifiers (contact email, hospital name and postal code) to prevent the risk of duplicate entries (the survey flow chart is shown in Supplementary Fig. 1).

Given the absence of patients or interventions in this study, ethical approval was not required. The data was anonymized and treated confidentially, and the participants were informed that their answers could be used for research purposes.

The survey was pretested with professionals matching the profile of the target participants (7 hospital acute care doctors from 2 different centers with a median completion time of 3 min 13 s). These physicians did not participate in the subsequent survey.

At the time of the study the national authority for emergency medicine - the Swiss Society of Emergency and Rescue Medicine - recognized 53 emergency departments in Switzerland. These centers were divided by this national society in categories according to the classification criteria for postgraduate training institutions established by the Swiss Institute for Postgraduate Medical Education (selected classification characteristics are shown in Supplementary Table 1). There were 16 category I centers, including the 12 Swiss level-1 trauma centers, of which 5 were university hospitals, and 37 category II centers. The invitation to participate in this survey was distributed through an email containing a link to the online questionnaire. It was sent to the medical education manager or an equivalent person in charge of all these EDs (e.g. medical director). To ensure the perceived legitimacy of the study, the invitation was sent from an identifiable institutional email address from our hospital. We described our identity, the aim of the study, the recruitment process, the median time of completion and the name of the center contacted.

The survey invitation and the first reminder were sent in English on March 3, 2025 and on March 26, 2025 respectively. The final reminder was sent on April 15, 2025 in both the center’s primary language (German, French or Italian) and English. A follow-up message to inquire about the reasons for not participating was sent on April 30, 2025 to nonrespondents. The survey was closed on May 7, 2025.

As 2 emergency departments were in the same city, operated under the same organizational structure and by the same medical manager, who provided grouped data for both centers, they were merged for the analysis. As a result, 52 centers were analyzed out of the 53 initially identified. The response rate was calculated under the assumption that all non-responding centers were eligible. Microsoft Excel was used for data curation and analysis.

## Results

### Response rate

**Fig. 1:**
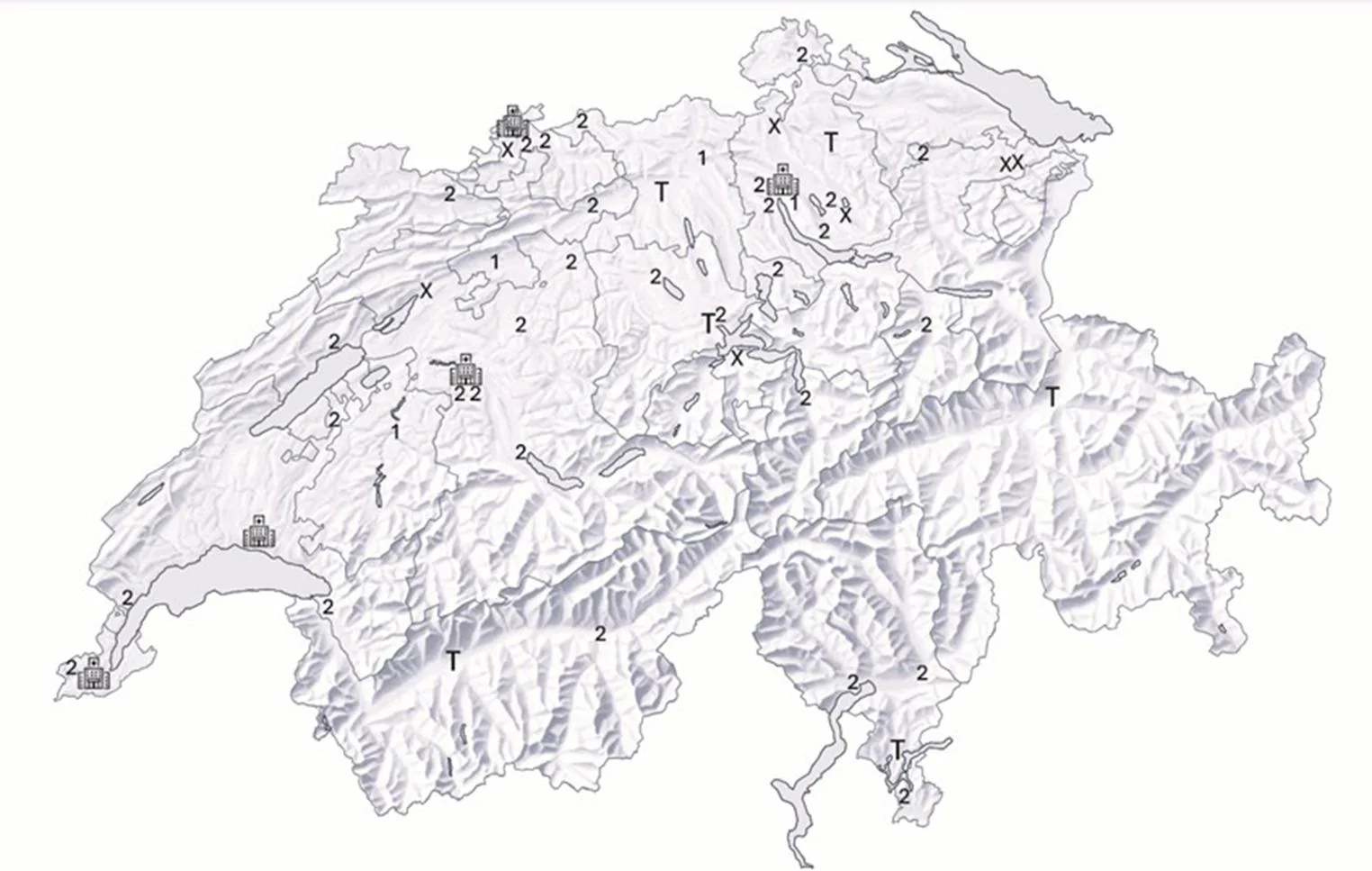
Geographical location of the emergency departments recognized by the Swiss Society of Emergency and Rescue Medicine. *Map of Switzerland (with the permission of the Federal Statistical Office, 2023 https://www.atlas.bfs.admin.ch/maps/13/fr/18055_75_3501_70/27876.html. The lines on the map indicate the boundaries between districts. The presence of symbols indicates the centers contacted for the study with the following correspondence: hospital representation: university hospital, T : respondent trauma center, 1 : category I non-trauma center, 2 : category II center, X : nonrespondents*

The response rate was 86.5% (45/52). The subcategory response rates were 93.8% (15/16) for category I centers (including 91.7% (11/12) for trauma centers and 100% (5/5) for university hospitals) and 83.3% (30/36) for category II centers. The respondent centers are distributed evenly across the country as shown in the geographic distribution of the contacted centers in Fig. 1. Half of the respondent centers reported between 20’000 and 39’999 ED visits per year (48.9%, 22/45), about a quarter (26.7%, 12/45) reported > 40’000 visits per year and the remaining (24.4%, 11/45) reported < 20’000 visits per year (the distribution by the number of visits per year is shown in Supplementary Fig. 2).

The response rate was higher in the second reminder that was written in the main language of the centers.

The median response time was 3 min 43 s.

The follow-up failed to elicit comprehensive responses from nonresponding centers. Only one of the last 12 contacted centers replied, indicating that its non-participation stemmed from a perceived lack of eligibility. After being informed of its eligibility, the center agreed to participate in the survey.

### VET access, use and protocol availability

Findings about VET access, use and protocol availability (for use and/or interpretation) are reported in Table 1. VET access, routine use and protocol implementation were 44.4% (20/45), 26.7% (12/45) and 26.7% (12/45), respectively. These rates, as well as the VET utilization and protocol availability among centers with access were greater in higher-level hospitals (category I, trauma centers, and university hospitals) compared with category II hospitals.

**Table 1:** Online survey quantitative findings

Online survey		Category I			Category II	Total	%
		All category I	Trauma center	University Hospitals			
Response rate	Initial (03.03.25)	6	4	2	8	14 (/52)	26.9%
	1st reminder (26.03.25)	3	2	0	6	9 (/38)	23.7%
	2nd reminder (15.04.25)	4	4	3	13	17 (/29)	58.6%
	Follow-up (30.04.25 to 07.05.25)	2	1	0	3	5 (/12)	41.7%
	Respondents/target population	15/16	11/12	5/5	30/36	45 (/52)	86.5%
Legal status	Public/respondents	9/15	7/11	4/5	13/30	22 (/45)	48.9%
Language	German	10	7	3	21	31 (/38)	81.6%
	French	4	3	2	6	10 (/10)	100%
	Italian	1	1	0	3	4 (/4)	100%
VET	Access	12	9	4	8	20 (/45)	44.4%
	Use	11	9	4	1	12 (/45)	26.7%
	Protocol	9	8	4	3	12 (/45)	26.7%
MHP	MHP for ED trauma	13	11	5	9	22 (/45)	48.9%
	MHP with CCT	12	10	5	9	21 (/22)	95.5%
	MHP with VET	11	9	4	2	13 (/22)	59.1%

CCT: Conventional Coagulation Tests, ED: Emergency Department, MHP: Massive Hemorrhage Protocol, VET: Viscoelastic Testing

### VET location

Concerning the location of devices, the majority were in the laboratory (70%, 14/20), followed by the operating room (40%, 8/20), and a minority were in the ED (10%, 2/20). The two centers with on-site use were university hospitals. No center reported another location for these devices. One center that mentioned the location in the laboratory specified that it was near the trauma bay and the results were displayed on the computers in the ED in real time.

### Type of device

ROTEM was the most used viscoelastic assay among respondent centers, reported by 18 out of 20 (90%). In contrast, TEG and ClotPro were each used by only one center (5% each), both of which were university hospitals. None of the centers reported using the Quantra device.

### Training

The survey was sent to medical education managers to assess the clinical training offered for these tools.

A small majority of centers that reported access to these techniques (55%, 11/20), and all category II centers, did not provide any specific training regarding the use and interpretation of viscoelastic tests. In category I centers no training was reported in 33.3% (4/12).

The reported educational formats were lectures (58.3%, 7/12), one-pagers (50%, 6/12), hands-on sessions (41.7%, 5/12), asynchronous e-learning (8.3%, 1/12), interactive e-learning (8.3%, 1/12) and a video on an open-access platform (8.3%, 1/12).

### Massive hemorrhage protocols

Approximately half of the centers had a massive hemorrhage protocol for emergency department use (48.9%, 22/45). Of these massive hemorrhage protocols, the majority used conventional coagulation tests (95.5%, 21/22) and a lower proportion used VET (59.1%, 13/22). All the massive hemorrhage protocols with VET also used conventional coagulation tests (59.1%, 13/22).

In the subgroup of category I centers, 86.7% (13/15) had a massive hemorrhage protocol. These protocols used conventional coagulation tests and VET in 92.3% (12/13) and 84.6% (11/13), respectively.

Of note, 2 category II centers reported VET used in their massive hemorrhage protocols. However, one of them did not report VET access in the above section. We contacted this center, which confirmed that they did not have access to VET for emergency department patients, but that the protocol was institutional and did include the use of these techniques.

### Comments

The comment section was completed by 7 centers. The most common remark (4/7) was that the center did not receive major trauma patients. One center specified the device’s location (in a lab near the trauma bay with remote real-time monitoring). Another noted that massive hemorrhage protocols included the use of these techniques, mainly interpreted by anesthesiologists; primarily because massively transfused patients were transferred to the operating room. Lastly, one center mentioned that a publicly available video supplemented the educational tools listed in the questionnaire.

## Discussion

### Key results

This study provides the first overview of viscoelastic assay access and use in Swiss emergency departments. The representativeness of the Swiss EDs is ensured as the centers are cataloged by the national authoritative society and because the study achieved a high response rate.

Access to these techniques was relatively common. However, their routine use remained limited and was mostly confined to high-level centers, particularly trauma and university hospitals. Use was generally protocol-driven. Devices were often not located within the emergency department, and ROTEM was the most frequently used system. Training was limited and heterogeneous, with the main obstacle being the absence of structured educational programs. Massive hemorrhage protocols largely reflected current guidelines, relying predominantly on coagulation-guided strategies, mainly through conventional coagulation tests, and to a lesser extent, viscoelastic testing.

### Strengths and limitations

The response rate was high (86.5%). It may be affected by confidentiality concerns and potential technical problems inherent to online-based surveys (e.g. email in spam folder, link access blocked by the recipient’s IT system). However, this format was chosen due to its cost-effectiveness, quick turnaround time, and minimal interviewer bias. During the recruitment process, only one center contacted us directly to find out if the survey was legitimate because of cybersecurity concerns. The population of this survey is the total target population so there is no risk of sampling bias. However, selection bias in the form of nonresponse bias is likely. Indeed, it is possible that the remaining 7 nonrespondent centers (including one trauma center) may have less interest, access or use of viscoelastic testing in their emergency department.

### Interpretation

Viscoelastic testing use in Swiss emergency departments seems positively correlated with centers providing a higher level of care (80% in university hospitals, 81.8% in trauma centers, 73.3% in category I and 3.3% in category II). This lower use correlated with the decreasing center category, which could be a target for an intervention. Indeed, a useful feature of a normal viscoelastic assay is its high negative predictive value regarding the need for the administration of blood products and thus could be potentially used as a triage tool, even in non-trauma centers [10, 13, 14].

VET was included in the massive hemorrhage protocol in a substantial proportion of centers that have such protocols (59.1%, 13/22) and was the standard of care in 60% (12/20) of centers that reported having access to them.

The utilization of such tests in the ED is reported in observational and interventional studies involving mainly trauma centers [8, 10, 13–18]. However, the prevalence of their use in clinical practice is unclear. A 2014 survey on a population of an international trauma congress with a response rate of 29% and 70% of participants from level-1 trauma centers has shown a 31.1% rate of “extended coagulation assays” use including VET and platelet aggregometry [19]. In contrast, our study, specific to emergency department setting with 24.4% (11/45) of participants from level-1 trauma-centers, found a 26.7%. routine use of viscoelastic testing. It is difficult to compare these two studies, but it is likely, given the different representation of trauma centers in the two populations, that the use of these tests has increased with time. In line with this interpretation, a 2015 survey with 6 large academic European level 1 trauma centers showed access and standard use for the treatment of traumatic bleeding of 66.7% (4/6) and 33.3% (2/6), respectively [20]. If we compare these results with the trauma centers of our 2025 survey, 81.8% (9/11) reported access and standard use of these techniques, suggesting a growing trend in their clinical applications with time. This fact is confirmed by a recent multicenter retrospective observational study in Queensland, Australia, that reported an increase in use of VET between 2019 and 2025, particularly in trauma, both in the ED and in the hospital settings [21].

The most frequently reported device in our population was the ROTEM (90%, 18/20), followed by the TEG (5%, 1/20) and Clotpro (5%, 1/20). Some multicenter studies have also noted a predominance of ROTEM devices [21, 22]. This information is interesting as these different devices perform the same type of analysis (i.e. clot dynamic with numerical and graphical values) and have a similar clinical performance, but their results cannot be directly compared [23]. It is reasonable to consider that coagulation metrics, such as VET readings, could hold similar importance in trauma care as other laboratory values like blood count, creatinine, or lactate levels. If this data is to be incorporated into our national trauma registry, standardizing using a single device nationwide could offer significant advantages.

The location of these devices was mainly outside the site-of-care (90%, 18/20), which may limit their usage frequency and hinder real-time result interpretation. One of the features often highlighted of these devices is their ability to be in the site-of-care [24]. However, their location is often not specified in the context of traumatic bleeding care in the ED. The aforementioned study in Queensland had shown a location in the ED for only 1 VET device out of the 39 reported in the 20 hospitals studied [21]. A study on the implantation of a ROTEM device in a level-1 trauma center in the intensive care unit for ED patients’ management revealed logistical issues to perform this test (staff availability, communication and sampling) [18]. Comparatively, a multicenter Spanish study in the operative setting has shown that the VET device was most frequently located in the site-of-care (87% in surgical or resuscitation unit), suggesting that the site-of-care is frequent in perioperative bleeding management [22]. It is therefore the authors’ opinion that we should further promote the on-site location of VET in the ED setting.

Finally, most centers that do have access to VET reported that their staff received no specific training on their use. This finding is in line with the perception of some authors studying VET and should motivate the development of dedicated training programs [8, 24–26]. Interestingly, a recent study on anesthesia clinicians’ perception of ROTEM found the need for training and difficulties in interpretation amongst the most reported comments [27]. In this study, 81% of respondents agreed that repetitive training would be beneficial. Education is most likely a critical determinant in improving the practice of these tools.

The percentage of massive hemorrhage protocol utilization (48.9%) in our study can be compared to a recent survey in the Netherlands (2025) that reported a 100% utilization rate of such protocols in massive transfusion, which should motivate the systematic implementation of these [28].

### Generalizability

This survey studied the Swiss healthcare environment and therefore included relatively small centers and a high proportion of private hospitals (51.1% of respondents). These results could thus not apply to other, more centralized, trauma systems in other countries. Nonresponse bias limits the generalizability of the findings, as centers that do not utilize these technologies may have been less likely to participate.

## Conclusion

This study provides the first national overview of viscoelastic testing in the context of severe trauma care in Swiss emergency departments. VET remains primarily available and used in major trauma centers, with limited on-site accessibility and heterogeneous training practices. Despite variable implementation of massive hemorrhage protocols, those in use generally adhere to current recommendations advocating goal-directed coagulation management. These findings highlight the need for broader access, standardized training, and consistent protocol integration to optimize the use of VET in emergency trauma care.

## Supplementary Information

Below is the link to the electronic supplementary material.

Supplementary file 1 (DOCX 52.0 KB)

## Data Availability

The data supporting this study are available upon request. The center and contact details are confidential and will not be shared.

## Abbreviations

ED: Emergency Department
ROTEM: Rotational Thromboelastometry
TEG: Thromboelastography
VET: Viscoelastic Testing
