# Supplementary material for: Multicenter survey on viscoelastic assays access and use in trauma in Swiss emergency departments
Source: Eur J Trauma Emerg Surg. 2026 Mar 2;52(1):76. doi: 10.1007/s00068-026-03092-w (PMC13668999; doi:10.1007/s00068-026-03092-w)
Supplement: Supplementary file 1 [file 68_2026_3092_MOESM1_ESM.docx]

Supplementary Table

**Table S1** Swiss emergency departments categories

| Emergency department characteristics | Cat. 1 | Cat.2 |
| --- | --- | --- |
| Visits (per year) | > 20’000 | > 8’000 |
| Anesthesiology and operating room 24/24h | + | + |
| Internal medicine and surgery 24/24h | + | + |
| Multidisciplinary intensive care | + | IMC or ICU |
| Radiology 24/24h | + | + |
| Interventional radiology 24/24h | + | - |
| Laboratory 24/24h | + | + |
| Gastroscopy/Coloscopy 24/24h | + | + |
| Interventional cardiology 24/24h | + | - |
| Bronchoscopy 24/24h | + | - |
| Stroke center | + | - |
| Head of training with a diploma in hospital emergency medicine; % of position | 100% | 60% |
| Deputy head of training with a diploma in hospital emergency medicine; % of position | 100% | 40% |
| Staff-member with a diploma in hospital emergency medicine for at least 2 shifts/day | + | - |
| Acute care nurses | 60% | 40% |
| Entire postgraduate training catalog | + | - |
| Structured specific postgraduate training (hours/week) | 2 | 0.5 |
| Research activity | + | - |

Selected characteristics used to classify centers in categories according to hospital capacities and metrics from the Swiss Institute for Postgraduate Medical Education. IMC: Intermediate Care, ICU: Intensive Care Unit.

Supplementary Figures

**Fig. S1** Online survey design

Flow chart of the survey with the 17 steps of which 9 were mandatory for all participants. CCT: Conventional Coagulation Tests, ED: Emergency Department, MHP: Major Hemorrhage Protocol, VET: Viscoelastic Testing.

**Fig. S2** Respondent centers capacity

|  |  |
| --- | --- |
| <20'000 | 11 |
| 20'000-39'999 | 22 |
| 40'000-59'999 | 8 |
| >60'000 | 4 |

Histogram showing the number of respondent centers (y-axis) distributed according to semi-quantitative categorization of emergency department visits per year (x-axis).
